# A tale of two bridges: Factors influencing career choices of trainee nursing associates in England: A longitudinal qualitative study

**DOI:** 10.1002/nop2.1266

**Published:** 2022-06-05

**Authors:** Rachel Louise King, Bethany Taylor, Sara Laker, Emily Wood, Michaela Senek, Angela Tod, Tony Ryan, Sally Snowden, Steven Robertson

**Affiliations:** ^1^ Division of Nursing and Midwifery, Health Sciences School University of Sheffield Sheffield UK; ^2^ Department of Undergraduate Nursing Winona State University Winona Minnesota USA

**Keywords:** education nursing, health workforce, nursing associates, staffing, United Kingdom

## Abstract

**Aim:**

The nursing associate role has created a new second‐level nursing role and provided an alternative route into registered nursing. For some, this provides a previously inaccessible opportunity for career progression. The aim of the study was to understand the factors that influence career choices of trainee nursing associates.

**Design:**

A longitudinal qualitative study of trainee nursing associate motivations, experiences and career aspirations.

**Methods:**

Semi‐structured interviews with trainee nursing associates from across England, UK, in February 2020 (*N* = 14) and March 2021 (*N* = 13). Diary data were also collected. Interview and diary data were analysed thematically. Reporting has followed COREQ guidelines.

**Results:**

Nursing associate training was viewed by some as a bridge into registered nursing. Role ambiguity led several to seek perceived security offered by the Registered Nurse profession. Those preferring to remain as nursing associates were keen to embed the bridging role between healthcare assistants and Registered Nurses, valuing a positive workplace culture.

## 1 INTRODUCTION

The nursing associate (NA) role was recently introduced to the health and social care workforce in England to provide a bridging role between unregistered healthcare assistants and Registered Nurses, filling a perceived skills gap and offering an alternative route into nursing (Health Education England, 2015). It is a generic role across all fields of nursing (adult, child, mental health and learning disability), providing care for people of all ages across a range of health and social care settings (NMC, 2018a). Previous research has explored the experiences of trainee nursing associates (TNAs) (Coghill, 2018a; Coghill, 2018b; King, Ryan, Wood, Tod, & Robertson, 2020a; Vanson & Bidey, 2019) and how qualified NAs are deployed in the workplace (Kessler et al., 2020a; Kessler et al., 2020b; Kessler et al., 2020c; Lucas et al., 2021b); however, little is known about the factors that influence their career choices.

## 2 BACKGROUND

Globally, healthcare assistants (HCAs) and those in equivalent roles, providing care and support in a wide range of settings, have often lacked opportunities for career progression. In England training for HCAs is recommended, but not mandatory (Health Education England, 2015) and there have been calls to support career development of this group (Health Education England, 2015; Snyder, Dahal and Frogner, 2018; Wakefield et al., 2009). The introduction of nursing associates (NAs), a new second‐level nursing role in England has been welcomed by individuals who would otherwise lack the opportunity for further training (Coghill 2018a, and 2018b and; King et al., 2020a,2020b), particularly those who, for financial reasons, or due to a lack of academic role models (Burnell, 2015), have been unable to follow their aspirations of entering the healthcare profession.

Many high‐income countries deploy second‐level nurses, such as enrolled nurses in Australia and New Zealand, and licenced vocational nurses or licenced practical nurses in North America, who provide fundamental care, undertake patient observations and support rehabilitation (Lucas et al., 2021a; The Health Foundation et al., 2018). The scope of practice of second level nurses varies globally, but generally they work under the supervision of Registered Nurses and are prepared at diploma level, compared with degree level nursing programmes (Lucas et al., 2021a). The NA role is a new second‐level nursing role in England and follows on from the second level “state enrolled nurse” (SEN) in the UK which was discontinued in the 1990s in a move towards an all‐graduate nursing profession (Glasper, 2016).

Several assumptions have been made about the NA role that half of NAs will transition to become Registered Nurses (RNs), that the transition to nursing would be smooth and that there would be lower attrition rates than other student nurse programmes (Health Education England, 2015). Evidence from a national evaluation of the first two pilot cohorts of TNAs (Vanson & Bidey, 2019) supports this first assumption, with almost 47% planning to apply to join a pre‐registration nursing degree programme. Furthermore, the majority of TNAs are motivated by local career progression, particularly the option of becoming Registered Nurses (Coghill, 2018b; King et al., 2020a,2020b). The role has been welcomed by organizations keen to grow their own workforce to fill nursing shortages (Kessler et al., 2020b, 2020c). TNAs are paid at band 3 of the NHS “agenda for change” pay scale (pay range £19,737 to £21,242) and join band 4 when qualified (pay range £21,892 to £21,157). Registered Nurses commence at band 5 (£26,970 to £30,615) and can progress to band 8a, earning up to £51,668 as advanced clinical practitioners (NHS Employer, 2020).

Barriers to the success of second‐level nursing roles include role ambiguity, intra and inter‐professional conflict and restrictions on practice (Lucas et al. 2021a). Furthermore, the importance of clarifying role identity and opportunities for career development have been raised by key stakeholders (Kessler et al., 2021), with some areas found to be less suitable for NAs such as intensive care, due to limitations in their scope of practice (Lucas et al., 2021b).

This study is situated within a larger mixed methods longitudinal TNA cohort study exploring motivations for undertaking training, experiences of training and career aspirations. This paper focuses on findings from the qualitative data relating to factors that have influenced career aspirations and choices.

## 3 THE STUDY

### 3.1 Design

This study has used a longitudinal qualitative design, collecting interview and diary data from the same TNAs over a 2 years period. This design enabled an exploration of how experiences and aspirations changed as they qualified and entered the workforce (Hermanowicz, 2013; Neale, 2020; Shirani and Henwood, 2015).

### 3.2 Method

In 2019, TNAs were recruited to a cohort (*N* = 154) via email (sent from five Higher Education Institution course leaders across England to current students) and social media (Twitter). A subgroup of the TNAs in the cohort were purposively sampled to this longitudinal qualitative study (after agreeing to be contacted for interview) to ensure diversity of location, gender, ethnicity, age and previous healthcare experience. Twenty TNAs were invited to participate by emailing a participant information sheet and consent form. They were offered a £10 shopping voucher as a thank you. A total of 14 TNAs from across England agreed to take part; three were male and 11 were female, two were Black, one Asian, and 11 were White British. They represented the North‐West, Yorkshire and Humber, East Midlands, West Midlands, London, South‐West, North‐East and South‐East of England. Eleven had previously worked as healthcare assistants/support workers and three did not have any previous healthcare work experience.

Semi‐structured interviews were undertaken at two timepoints (February 2020 and March 2021) providing flexibility, reciprocity and allowing the interviewer to improvise and explore key points using a natural flow of conversation (Kallio, Johnson & Kangasniemi, 2016). Interviews followed the six stages outlined by Ritchie, Lewis, McNaughton Nicholls, & Ormston (2014) and were undertaken via telephone or video call (Zoom or Google meet), at a time convenient to participants. Thirteen agreed to be interviewed in year two (one did not respond). The non‐responder was female, white British. This excellent retention rate in year two was promoted through the careful building of rapport during the year one interview (Hermanowicz, 2013). Interviews lasted between 21 and 50 min (average 34 min). Three members of the research team (RK, SR and SA); one male, and two female, with extensive post‐doctoral experience of undertaking qualitative research conducted the one‐to‐one interviews. Two of these researchers were also Registered Nurses.

The interview topic guide (see Box 1) was developed from key policy documents and areas of importance to TNAs identified in previous research (King et al., 2020a,2020b). It was piloted with two TNAs to ensure clarity of the questions. The year two interviews explored the same broad themes as the first year interviews in addition to revisiting and updating previous understanding in an iterative way (Neale, 2020). Interviews were audio‐recorded, transcribed and anonymized.

Participants were also sent a diary prompt every three months over a two year period with a link to an electronic google form which collected the entries (see Box 2). Diary data have been shown to be useful in capturing data around work–life identity issues (Radcliffe, 2013). A total of 16 diary entries were submitted between February 2020 and April 2021, completed by five participants. Collecting data over time provided insights into how the experiences of participants unfolded during their training, and a depth of understanding about shifting career aspirations (Neale, 2020). Reporting has followed the Consolidated Criteria for Reporting Qualitative Research (COREQ) guidelines (Tong, Sainsbury, & Craig, 2007).

### 3.3 Analysis

Data were analysed thematically using Braun and Clarke’s (2020) most recent iteration of their six‐step framework as a guide (Box 3), attending to the dynamic, recurring and cumulative significance of longitudinal qualitative data (Neale, 2020). Quirkos^©^ computer assisted qualitative data analysis software (CAQDAS) was used to support this stage of analysis. Initial coding was completed independently by [RK]. Further categorizing and collapsing of codes into themes was completed jointly by [RK, SR and SL]. Final confirmation of themes was agreed in discussion and agreement with the whole research team.

Rigour has been enhanced by undertaking recognized methods of data collection and analysis, providing a detailed description of the study design and using researcher and source triangulation (Ritchie et al., 2014). Triangulation of sources involved data collection using both interviews and diaries, ensuring a depth of understanding of the experiences of participants (Ritchie et al., 2014). Furthermore, triangulation of analysis was achieved when data were analysed by one researcher, checked by another and discussed and refined in the wider research team (Ritchie et al., 2014). Trustworthiness was further enhanced by attending to discrepancies in the data (Lincoln and Guba, 1985). Pragmatic data saturation was reached through the generation and contextualization of categories (Low, 2019).

### 3.4 Ethical considerations

TNAs were sent an information sheet and consent form via an invitation email. Written consent was gained (via email) prior to the interview and diary data collection. Participants were reminded that they could withdraw at any time, although anonymized data incorporated in the analysis could not be removed. Confidentiality was maintained; however, participants were advised that if they disclosed any harm to themselves or others this would be reported to the relevant authority. Ethical approval was gained from the University of Sheffield Research Ethics Committee [Ref: 026355].

## 4 RESULTS

This paper presents the findings related to the career choices of TNAs. In the first year of data collection, six TNAs stated they would be keen to undertake RN training at the earliest opportunity, with five stating they would rather stay as an NA and three remaining undecided (see Table 1). In the second year, most participants were consistent in their choice of career trajectory; however, one participant who had been keen to stay as an NA had changed their mind to being undecided about their future career path (TNA 6), and one who had initially planned to pursue RN training altered their choice and decided to remain as an NA (TNA 9).

**TABLE 1:** Career choices of TNAs/NAs in year one and year two

Participant	Year one career plan RN (top up to RN) NA (stay as NA) U (undecided)	Year two career plan RN (top up to RN) NA (stay as NA) U (undecided) X (non‐respondent)
TNA 1	RN	RN
TNA 2	RN	RN
TNA 3	RN	RN
TNA 4	NA	NA
TNA 5	NA	X
TNA 6	NA	U
TNA 7	U	U
TNA 8	NA	NA
TNA 9	RN	NA
TNA 10	RN	RN
TNA 11	U	RN
TNA 12	U	U
TNA 13	NA	NA
TNA 14	RN	RN

Factors that influenced participant career choices are discussed in relation to reasons for pursuing RN training (theme one) and reasons to remain as an NA (theme two). In theme one the RN role is viewed as the end point for some TNAs, and for others it is perceived as a more attractive option for those who were disillusioned with the NA role. In theme two, some participants who chose to remain as NAs were invested in embedding the new role, others enjoyed the positive experience of feeling valued in the workplace, and some faced barriers to accessing RN training.

### 4.1 Theme 1: Factors influencing the decision to pursue a registered nursing career

Several participants planned to pursue further training to become RNs. Some who expressed a desire to “top‐up” their training perceived the TNA programme as an affordable route into registered nursing. They viewed it as an opportunity to achieve their goal of becoming RNs; a way for them to fulfil their aspirations, some with specific specialities in mind. Others had become disillusioned with the role ambiguity and subsequent confusion associated with the NA role and therefore saw the RN role as a more attractive option, perceived to have a clearer professional identity and upward career pathway.

#### 4.1.1 NA training as a route to achieving RN status

For most participants who were keen to complete the RN degree, the goal from the beginning had been to use this opportunity to become RNs. NA training offered a previously inaccessible route for them, mainly due to financial constraints. Some had clear ambitions of where they hoped to work within the nursing workforce, and NA training was a stepping stone towards achieving that goal. For most, that career plan was unchanged in year two.

“It just made sense in so many ways. It meant I could continue my journey of becoming a Registered Nurse, but do it in a way that was better designed for those that had commitments financially”. TNA 14 Yr 1 interview

“I want to progress. If I could be an advanced clinical practitioner, I would” TNA 1 Yr 1 interview

“I’m going to do the top‐up for definite because that was always the plan was to do top‐up and do my band five”. TNA1 Yr2 interview

One participant who had changed her mind across the 2 years had been undecided in year one. However, in the second interview she explained that she had come to recognize the limitations of the NA role in terms of career development:

“A lot of the things I want to do, you can’t do as a [band] four. I was always interested in a discharge coordinator, or research role, I wouldn’t have minded that”. TNA11 Yr2 interview

For some participants, there was frustration about the lack of options to progress as an NA. Despite seeing clear similarities to the junior RN role, with few distinctions, there would be little opportunity for promotion without further training:

“There’s very little actual difference between a nursing associate and a nurse at band five, generally… It’s only once you go to band six and you start specialising…that’s only the real big changes. The other little bit is IVs [intravenous injections], why wouldn’t you want to get paid more to do a little bit of IVs?” TNA2 Yr2 interview

“I feel like the NA role, there’s a glass ceiling. And there’s not really anywhere for me to go up, it’s sideways, so the only way for me to kind of achieve what I want to achieve, and have flexibility to kind of drop my hours, or spend time with my child, is to go onto the next level”. TNA 3 Yr2 interview

Clearly, NA training has opened up opportunities for those who would otherwise find it difficult to access the RN qualification; providing an alternative route into the nursing profession; an important goal of the widening participation agenda, and a mechanism to move up the career ladder.

#### 4.1.2 Disillusioned with the NA role

In addition to viewing NA training as a route into nursing, others talked about choosing to undertake an RN “top‐up” programme to escape the challenges associated with the NA role. They described experiences of role ambiguity and conflict and felt that these problems would be overcome by joining the more established registered nursing profession, as noted by one participant in his interview and diary entries:

“I think that a lot of people have got frustrated with the role because nobody knows what the role is…Whereas everyone knows what a nurse can do, and there’s a lot of politics around a nursing associate, can’t do this but can do that”. TNA2 Yr2 interview

“The ward were not expecting me and seemed lost to have me there… she then said to each one ‘Please can you have [name of TNA] and he will support you, each one in turn then said no and came up with an excuse of ‘No, pass him on to X', like an unwanted puppy. This made me feel very small and unwanted… It does not feel like a team working together to promote patient care”. TNA 2 diary

Another participant reflected on her frustrations at not being used to her full potential. She also described how others in their cohort were disillusioned with their experiences. In managing role ambiguity some TNAs developed strategies to educate those around them.

“It’s just really difficult going to work, and kind of having all this knowledge, and this experience, and kind of being pushed in the corner, if you like, kind of being an afterthought…People have used it [as a route into nursing], and just got straight off at the next stop, and you know, we wouldn’t have done that if the role was valued… it’s not worth doing all the donkey work, when somebody is sat next to you being paid more than you, and being respected more than you.” TNA 3 Yr2 interview

“I take a Nurse Associate pack with me on every placement, that I have put together myself. It holds copies of The Standards of Proficiency for Nursing Associates (NMC, 2018a) and The Code (NMC, 2018b) and other relevant information. It also holds information for others about our role as still there are so many that don’t understand it”. TNA 12 diary

The experience in the workplace was an important factor in influencing TNA career choices. Role ambiguity and the subsequent conflict experienced in work settings led several participants to become disillusioned with the NA role. However, others were keen to remain in the NA role, and the factors influencing those choices are discussed in the next section.

### 4.2 Theme 2. Factors influencing the choice to remain as a nursing associate

Several participants described their plans to remain in the NA role. In the first year of interviews five participants were planning to remain as NAs; however, in the second interviews one had changed their mind and had chosen to pursue the route into registered nursing. Factors influencing the choice to remain as an NA included investment and passion to promote and embed the new role, and a positive workplace culture where they had a feeling of belonging in a team that provided support and valued the role. Others faced barriers to accessing RN training.

#### 4.2.1 Investment in the NA role

Some participants had embarked on the NA training as they were keen to develop beyond the HCA role, but had no aspirations to pursue RN training. They described being content with the scope and opportunities offered by the NA role and were keen to embed it in the workplace. Other TNAs valued the breadth of opportunities offered by the generic NA role, which enabled them to work across the four fields of nursing, adult, child, mental health and learning disabilities:

“At this present time I want to become a more embedded nursing associate as opposed to everybody keeps saying to me go straight onto the Band 5, that’s not what I did it for initially, I’ve got no intention of doing Band 5, it was just getting out there, doing a role that I can cope with, be happy with, and actually enjoy” TNA 5 Yr1 interview

“I think the role of NA actually gives you a lot more flexibility…I think if people want the Registered Nurse role, that’s up to them. But I think possibly I could get a lot more experience and I’d fit in better as an NA. Because we’re generic aren’t we. So if I want to move, it’s a lot easier than if I was say a mental health nurse” TNA 8 Yr2 interview.

The variety of career opportunities afforded by the generic role influenced some to choose to stay in the NA role. However, this was made easier when they felt valued and supported through a positive workplace culture.

#### 4.2.2 Positive workplace culture

For some who chose to remain in the NA role, the key influencing factor was that they felt they had a secure position in their healthcare team and they perceived that the role was valued by their colleagues:

“I’m just so happy where I am that I don’t want to come out of that and do something I don’t want to be doing… and one of the band sixes came over and she actually said to me, [name], I know I’m not your mentor, but I’m so proud of you”. TNA 4 Yr2 interview

“And people were instantly respecting my knowledge, my skill. I had others who had been in the district for a long time coming to me for advice about some things, and it made me feel greatly appreciated.” TNA13 Yr2 interview

For one NA, the feeling of being a valued member of the team clearly provided a positive experience of the role. She also reflected on the difference her training had made to her practice, compared to her previous HCA role, describing how she effectively assessed and managed an acutely unwell patient:

“I absolutely feel my responsibilities and accountability are very important and this has come about because of my training. New team members have said they are very pleased to have me there because I am reminding them of the importance to remain current with their evidence‐based practice… I am loving my new role!” TNA 12 diary

“As I was waiting for the paramedics to arrive I was performing my ABCDE checks, I remember thinking to myself the patient smells really nice…I remember reading about Diabetic ketoacidosis (DKA) and that a person can smell sweet. I took his blood sugar reading and it was very high… It was a very important event for me as I do feel that I took that step up from Healthcare Assistant to Nursing Associate”. TNA 12 diary

One participant who had initially described her plan to undertake RN training, had changed her mind in the year two interview. She explained how much she enjoyed her work and felt valued in the service she provided, in addition to reaching capacity in terms of academic training:

“I’ve, kind of, done with all of that now, so maybe, I’ll just stick with what I’m doing… I just, I feel I’m making a difference to people’s lives and I love being able to advocate for people to make sure that, you know, they’re dying with dignity and they’ve got all of the support they need and it just makes me feel good, I love It.” TNA 9 Yr2 interview

For those who chose to remain as NAs it was important for them to feel they were making a difference as valued members of their teams; contributing to person‐centred safe and effective care. This was enabled through a positive workplace culture.

#### 4.2.3 Barriers to accessing RN training

Participants described other reasons for staying in the NA role, in terms of the barriers to accessing further training. One TNA felt that, although she would have loved to progress to become an RN, she was too old. However, when interviewed a second time she said that many people had encouraged her to pursue the transition to RN, so she was then undecided. Furthermore, a lack of development opportunities led some to remain as NAs, with a lack of support from their employers to undertake the RN training:

“If I was younger I would dearly love to go for it [RN training], but it’s my age, I’m 58 so by the time I’ve finished this and I get my qualification, which is next year I’ll be 59.” TNA 6 Yr 1 interview

“I wanted to do my nursing, but then they changed the goal posts to say it was then an 18 months top up, and because I was a mature student it was a bit disheartening towards the end when they said it was a longer top up. But I am absolutely loving it.” TNA 11 Yr1 interview

Despite the delay in accessing opportunities, this participant changed her mind from being undecided to pursuing the RN training. There was a sense among some participants that employers preferred to keep NAs in that role and were therefore not offering the top‐up opportunities. The COVID‐19 pandemic also caused delays in completion of NA programmes and subsequent “top‐up” opportunities, leading to uncertainty and anxiety among participants:

“One of the drawbacks at the moment is my [employer] not offering that top‐up and I think it’s because they want to keep us at band four.” TNA 12 Yr 1 interview

“It was soul destroying that it [training] was put on hold at the time it was, ’cause we just wanted the end. We were told there was going to be a six‐month delay…due to Covid” TNA 11 Yr2 interview

Participants highlighted the tensions between individual career aspirations and organizational workforce planning. These tensions and frustrations were further impacted by delays caused by the global COVID‐19 pandemic.

## 5 DISCUSSION

The career trajectory of participants in this study presents “a tale of two bridges.” The first is a bridge *into* registered nursing for those whose goal is to top‐up their NA training to the degree level RN qualification, bypassing the obstacles they previously faced. Some never viewed the NA role as the end point, but as a bridge to their destination of RN, others had become disillusioned with the challenges they faced in their new role. The second is a bridge *between* the two roles of HCA and RN, a position which some participants were keen to embed in their practice settings, committing to educating wider stakeholders on the scope of the NA role, and valuing their new knowledge, skills and opportunities.

Affordable career development was a significant influencing factor for both those choosing to stay as NAs and those hoping to become RNs. Several participants had financial commitments such as mortgages and dependents which had previously prevented them from stepping out of work into higher education. The funding offered by TNA programmes was essential to achieving their goals and ensuring widening participation; consistent with previous studies which found that NA training provided an affordable route into nursing and an opportunity for career development (Coghill 2018a, 2018b; King et al., 2020a,2020b; Vanson & Bidey, 2019). This is an example of where the widening participation agenda has benefited mature students, as well as those straight from school (Boeren & James, 2017; Burnell, 2015), providing a strategy for employers to enhance staff retention by “growing their own” nursing workforce (Health Education England, 2020). In this way, the NA training programme has provided a crucial opportunity for the upward career movement of experienced HCAs called for by previous researchers (Health Education England, 2018; Snyder et al., 2018).

For some participants, role ambiguity and conflict led to unpleasant working environments and a desire to move into the more secure, well‐known role of the RN. This is consistent with findings from the national TNA evaluation (Vanson & Bidey, 2019) which identified the negative impact on well‐being resulting from role ambiguity and lack of acceptance by colleagues. Similarly, Lucas et al., (2021a) found that second level nurses have been plagued by both role ambiguity, and intra‐ and inter‐professional conflict over the years. NA stakeholders in particular report a lack of a clear NA job descriptions and a requirement to jump between roles (Lucas et al., 2021b). Furthermore, King et al., (2020a) found that TNAs themselves were unsure of the scope of their role, adding to the confusion experienced by wider healthcare teams. Studies exploring qualified NA roles have shown that they are beginning to develop a distinct scope of practice and in some cases are meeting little inter‐professional resistance (Kessler et al., 2020b, 2020c).

The COVID‐19 pandemic has exacerbated the lack of role clarity, with some TNAs and NAs extending their scope of practice, others being underutilized, and several experiencing delays in their training (King et al., 2021). The current study reveals that role confusion and conflict can influence some to seek opportunities to enter the registered nursing workforce, perceived as having a more stable professional identity. Future research should evaluate strategies that can enhance a positive workplace culture for TNAs and NAs.

Healthcare workforce shortages have increased during the pandemic (Anderson et al., 2021; Azzopardi‐Muscat, 2020, Heath et al., 2020), and TNAs have faced concerns about safety, and delays to training and development (King et al., 2021). This study identifies how a positive workplace culture, one in which TNAs feel supported and valued, is important to participants and crucial to retaining staff at the NA level. Similarly previous research on the reasons for student nurse attrition found that lack of support and unmet expectations were important contributors (Health Education England 2018). Those who chose to remain in the NA role talked about making a difference and reflected on times when they felt valued through strong leadership and a positive workplace culture. These have previously been found to be key elements to optimizing professional development in nursing (King et al., 2020b), as opposed to a lack of support which can lead to demoralization in the workforce (Senek et al., 2020).

Finally, some participants faced barriers to progression, including uncertainty about embarking on further intensive training, a factor also reflected in previous research (Kessler et al., 2020c). Some felt that employers were withholding development opportunities to fit with their preferred service model, and this is reflected in previous cases where employers have insisted on NAs staying in their role for a period prior to undertaking RN training (Lucas et al., 2021b; Kessler et al., 2020b).

## 6 LIMITATIONS

Although recruitment to this study achieved diversity in terms of age, geographical location and previous work experience, the majority of participants were white British. Future recruitment to TNA/NA workforce studies should aim to increase the ethnic diversity of participants. A sample of 14 TNAs were included in the study, with 13 completing the second interviews after 1 year. Small sample sizes are usual in qualitative studies, allowing for depth of understanding. Transferability has been enhanced by including TNAs from across England, from a range of settings, in different age categories and with a variety of previous work experiences.

## 7 CONCLUSION

Career choices of TNAs are based on a number of factors and tensions may occur when personal career aspirations fail to align with workforce plans. The findings have implications for Registered Nurses, employers and educators. Employers and educators should work together to enable TNAs to pursue their preferred career pathway, addressing key issues raised in this study. Participants value opportunities for career development but crucial to their job satisfaction is feeling valued and working to minimize role ambiguity as the role is embedded in the workplace. It is therefore important that Registered Nurses and the wider healthcare team endeavour to acknowledge the workplace identity, value and scope of practice of NAs in health and social care settings.

## AUTHOR CONTRIBUTIONS

All authors planned the study and contributed the background literature. RK, SR and BT undertook the interviews. RK, BT, SR and SL analysed the data. All authors read and approved the final manuscript.

## CONFLICT OF INTEREST

No conflict of interest.

## Data Availability

The data that support the findings of this study are available from the corresponding author upon reasonable request.
